# An isoelastic monoblock cup versus a modular metal-back cup: a matched-pair analysis of clinical and radiological results using Einzel-Bild-Röntgen-Analyse software

**DOI:** 10.1007/s00402-023-05058-8

**Published:** 2023-09-23

**Authors:** Yama Afghanyar, Jens Hendrik Möller, Felix Wunderlich, Jens Dargel, Philipp Rehbein, Erol Gercek, Philipp Drees, Karl Philipp Kutzner

**Affiliations:** 1 Department of Orthopaedics and Traumatology, University Medical Centre of the Johannes Gutenberg-University of Mainz, Mainz, Germany; 2 Department of Orthopaedics and Traumatology, St. Josefs Hospital Wiesbaden, Wiesbaden, Germany

**Keywords:** Hip, THA, Cup, Monoblock, Modular, Vitamin E

## Abstract

**Introduction:**

Bone preservation and long-term survival are the main challenges in cementless total hip arthroplasty (THA). A good bone stock is especially important for adequate anchorage of the cup in revision cases. However, the optimal acetabular cup design for preserving good bone stock is still unclear. We aimed to compare clinical outcome, radiological alterations, migration, and wear at mid-term for two different cup types.

**Materials and methods:**

This retrospective matched-pair study was performed using the data for 98 THA cases treated with a monoblock cup composed of vitamin E-blended highly cross-linked polyethylene (VEPE; monoblock group) or a modular cup composed of a highly cross-linked polyethylene (HXLPE) without an antioxidant (modular group). Clinical results were evaluated using the Harris Hip Score (HHS). The obtained radiographs were analyzed for radiological alterations, migration, and wear using Einzel-Bild-Röntgen-Analyse (EBRA) software.

**Results:**

The mean follow-up duration was 73.2 ± 19.2 months (range: 32–108 months) and 60.5 ± 12.2 months (range: 20–84 months) in the monoblock and modular groups, respectively. HHS improved to 95.7 points in the monoblock group and 97.6 points in the modular group, without significant differences (*p* = 0.425). EBRA measurements were obtained in all cases. Acetabular bone alterations were not detected on radiological assessments. Mean cup migration was 1.67 ± 0.92 mm (range: 0.46–3.94 mm) and 1.24 ± 0.87 mm (range: 0.22–3.62 mm) in the monoblock and modular groups. The mean wear rate was 0.21 ± 0.18 mm (range: 0.00–0.70 mm) and 0.20 ± 0.13 mm (range: 0.00–0.50 mm) in the monoblock and modular groups. Both migration and wear pattern showed no significant differences (*p* = 0.741 and 0.243). None of the cases required revision surgery, yielding an implant survival rate of 100% in both groups.

**Conclusion:**

The isoelastic press-fit monoblock VEPE cup and modular metal-back HXLPE cup showed equivalent mid-term wear and cup migration. Long-term studies are required to determine the effects of modularity, isoelasticity, and polyethylene stabilization with vitamin E on cup loosening and survival rates.

## Introduction

Bone preservation and long-term survival are the primary challenges in cementless total hip arthroplasty (THA), especially in the light of the upcoming demographic changes and the predicted increase in revision surgeries in the future [1]. Subsequent aseptic loosening of the cup due to osteolysis and stress shielding of the surrounding bone remains the main reason for long-term complications [2–4]. In revision cases, a good bone stock is mandatory for sufficient anchorage of the cup. However, the optimal acetabular cup design to preserve good bone stock remains a topic of debate.

Modular metal-back cups offer a stiff and solid frame with high primary stability using an equatorial press-fit technique. The main advantage of these cups is their modularity, which allows easy placement and replacement of different inlays and inlay designs, if necessary. However, the backside wear caused by micromotions between the polyethylene and the inside of the metallic cup has been found to be a problem [5]. Additionally, the higher rigidity of the cup in comparison with the bone may lead to increased stress shielding [6].

The concept of isoelastic monoblock cups was proposed to reduce wear and eliminate the associated risk of acetabular osteolysis [7, 8]. Although this approach permits the use of thicker polyethylene and prevents backside wear, an elastic modulus closer to that of bone leads to superior load transfer onto the surrounding bone, potentially decreasing stress shielding in the long term [9].

The development of wear-resistant polyethylene has been a topic of major importance in the last few decades. Long-term results have demonstrated superior wear and implant survival for highly cross-linked polyethylene (HXLPE) in comparison with ultra-high molecular weight polyethylene (UHMWPE) [10, 11]. Since cross-linking decreased the wear, this process was associated with a reduction in fatigue strength [12]. To address this problem, vitamin E has been used in a proprietary diffusion process with polyethylene to prevent oxidative degradation and maximize strength and wear resistance [13–15].

Overall, these advancements led to the hypothesis that a vitamin-E-blended highly cross-linked polyethylene (VEPE) monoblock cup would show lower wear and migration rates than a modular metal-back cup with HXLPE, potentially resulting in lower revision rates and improved survivorship. Therefore, this study aimed to compare clinical outcomes, radiological alterations, migration, and wear between both cup types.

## Materials and methods

This retrospective matched-pair study was performed using in-hospital data for 98 cases of THA performed with 2 different cementless cups. The groups were matched from an overall study population of 258 patients by sex, age, body mass index (BMI), and American Society of Anesthesiologists (ASA) classification.

**Fig. 1:**
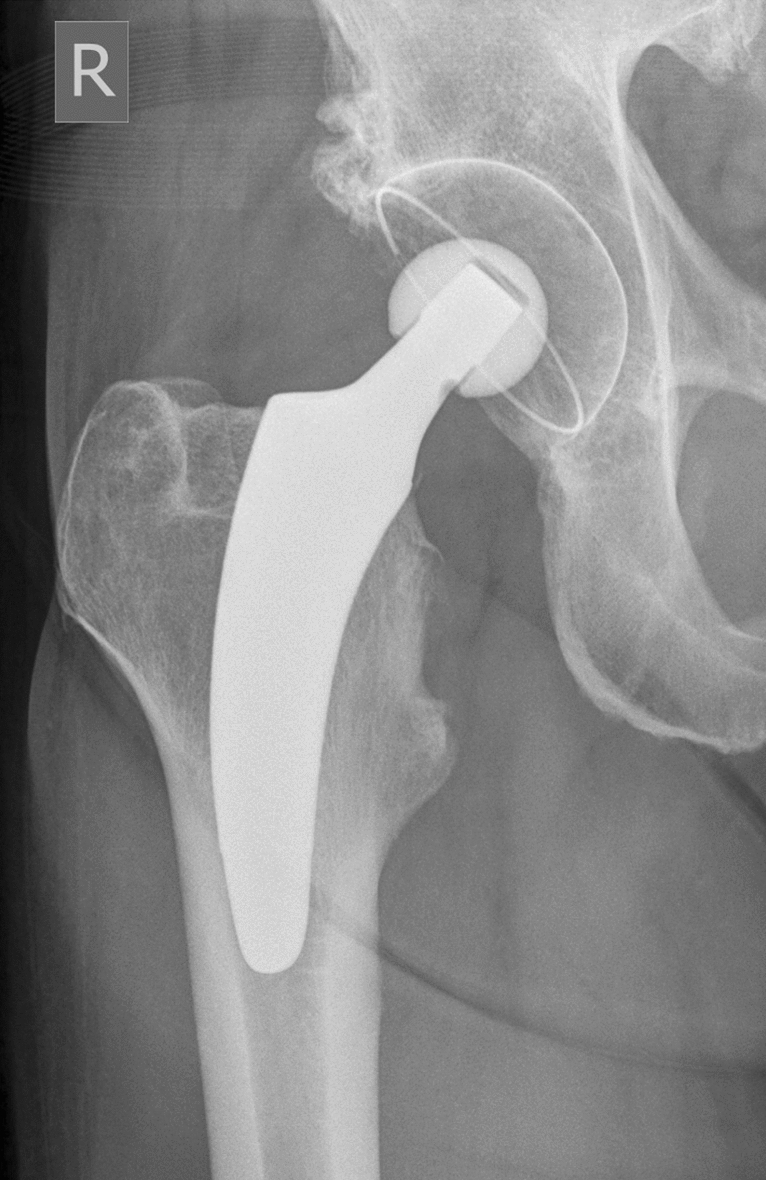
The RM Pressfit vitamys cup with an optimys stem (Mathys Ltd., Bettlach, Switzerland)

**Fig. 2:**
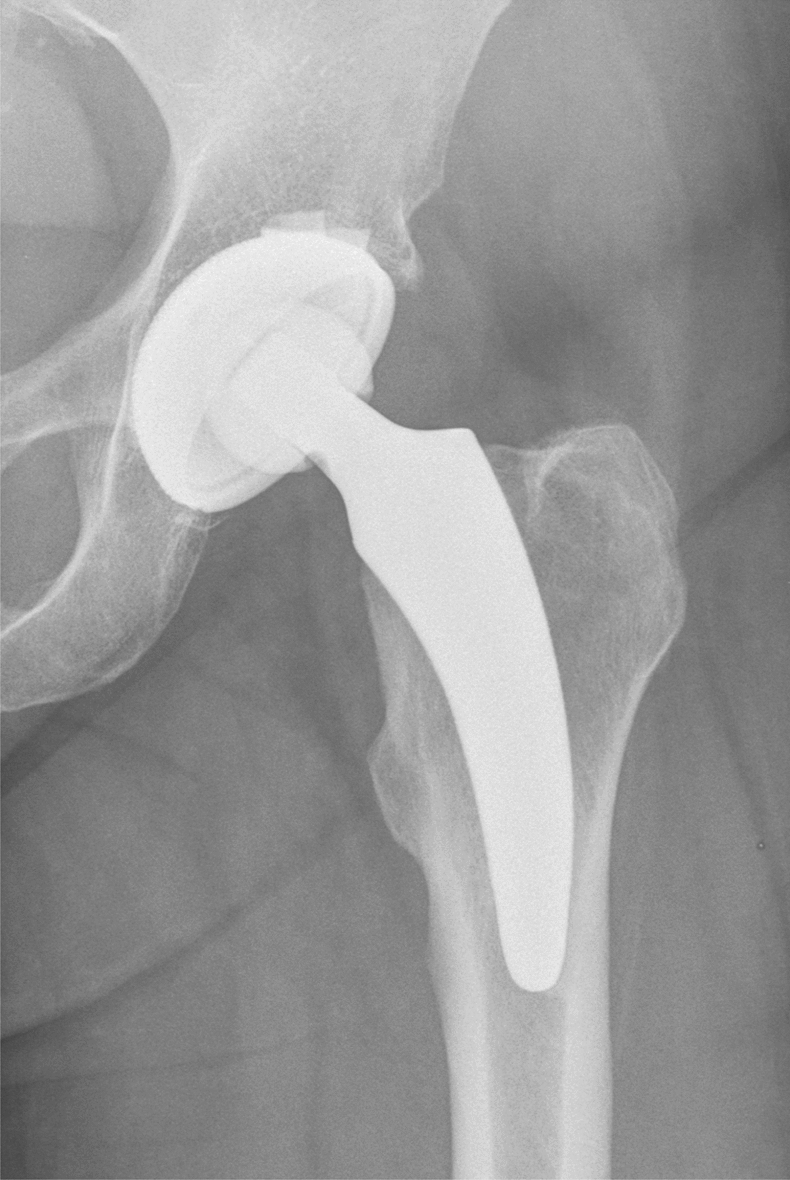
The Fitmore cup (Zimmer Biomet, Warsaw, USA) with an optimys stem (Mathys Ltd., Bettlach, Switzerland)

In the monoblock group, the RM Pressfit vitamys cup (Mathys Ltd., Bettlach, Switzerland) (Fig. 1) made of VEPE was implanted in 49 patients. This cup has an isoelastic titanium coating to mimic the elastic properties of natural bone. It also has a flattened pole and achieves primary stability by equatorial press-fit. Secondary stability was achieved through a bony ongrowth. In the modular group, the Fitmore cup (Zimmer Biomet, Warsaw, USA) (Fig. 2), a metal-back cup with a stiff titanium shell and a flattened pole, was implanted in 49 patients. In these patients, primary stability is achieved with an equatorial press-fit and additionally with two sharp-edged fins. The multilayer titanium mesh, which has a rigid porous structure, promotes secondary osteointegration. The liner is fixed within the metal shell using a snap-fit mechanism. The liner in all cases was Durasul® (Zimmer Biomet, Warsaw, USA), which is composed of HXLPE without antioxidants.

The cup design used for implantation depended on surgeon’s preferences and was not based on specific criteria. The cups in both groups were combined with cementless stems and ceramic heads supplied by different manufacturers.

The inclusion criteria were osteoarthritis of the hip and patient age over 18 years. Patients with posttraumatic osteoarthritis, bone metabolism disorders, or severe cardiovascular diseases were excluded. All surgeries were performed with a minimally invasive anterolateral approach in the supine position, and all patients were operated on by experienced consultant surgeons. Full weight-bearing under the guidance of physiotherapists was allowed starting the day of surgery.

All patients were informed about the study and gave their verbal and written permission to participate prior to inclusion. All procedures were performed in accordance with the 1964 Helsinki Declaration, and institutional ethics approval was obtained (2020-1851-evBO).

Clinical and radiological follow-up assessments were performed preoperatively, immediately after the operation, and then 6 weeks, 1 year, 2 years, and 5 years postoperatively.

Clinical outcomes were evaluated by obtaining the Harris Hip Score (HHS). Furthermore, complications and revision surgeries were documented. Additionally, standard anterior–posterior radiographs of the pelvis were acquired and analyzed for radiological alterations like lucent lines and osteolysis in the zones described by DeLee and Charnley [16]. In addition, acetabular cup migration and polyethylene wear were measured using the Einzel-Bild-Röntgen-Analyse (EBRA) software (University of Innsbruck, Innsbruck, Austria). All measurements were performed by the same observer to avoid inter-observer deviation.

The statistical analyses were conducted using SAS software 9.4 (SAS Institute, Cary, North Carolina, USA). All analyses were performed using standard descriptive statistics, including mean ± standard deviation (SD) and range. Differences among matched patients were evaluated using paired *t* tests, and Wilcoxon signed-rank tests were used for non-normal data. For statistical significance, a *p* value < 0.05 was considered.

## Results

The mean age of the patients undergoing THA was 66.8 ± 7.3 years (range: 33–78 years), and the procedures were performed for various indications. Detailed baseline demographic characteristics and diagnoses are illustrated in Table 1. The mean follow-up duration was 73.2 ± 19.2 months (range: 32–108 months) for the monoblock group and 60.5 ± 12.2 months (range: 20–84 months) for the modular group.

**Table 1:** Baseline demographic data

	Monoblock group	Modular group	*p* value
Number of patients (*n*)	49	49	
Mean age at operation (years) (range)	67.1 (50.7–78.7)	66.6 (33.4–75.3)	0.347
*Sex* (*n*) (%)			0.686
Female	25 (51.0)	27 (55.1)	
Male	24 (49.0)	22 (44.9)	
Mean BMI (kg/m^2^) (SD)	26.8 (4.03)	26.1 (3.41)	
Median follow-up (years) (range)	79 (32.0–108.0)	60 (20.0–84.0)	< 0.0001
*Diagnosis* (*n*) (%)			0.337
Primary osteoarthrosis	47 (95.9)	43 (87.8)	
Hip dysplasia	1 (2.0)	3 (6.1)	
Necrosis	1 (2.0)	3 (6.1)	
*ASA grade* (*n*) (%)			0.6993
Grade 1	4 (8.2)	2 (4.1)	
Grade 2	40 (81.6)	42 (85.7)	
Grade 3	5 (10.2)	5 (10.2)	

*ASA* American Society of Anesthesiologists, *BMI* body mass index, *SD* standard deviation

**Fig. 3:**
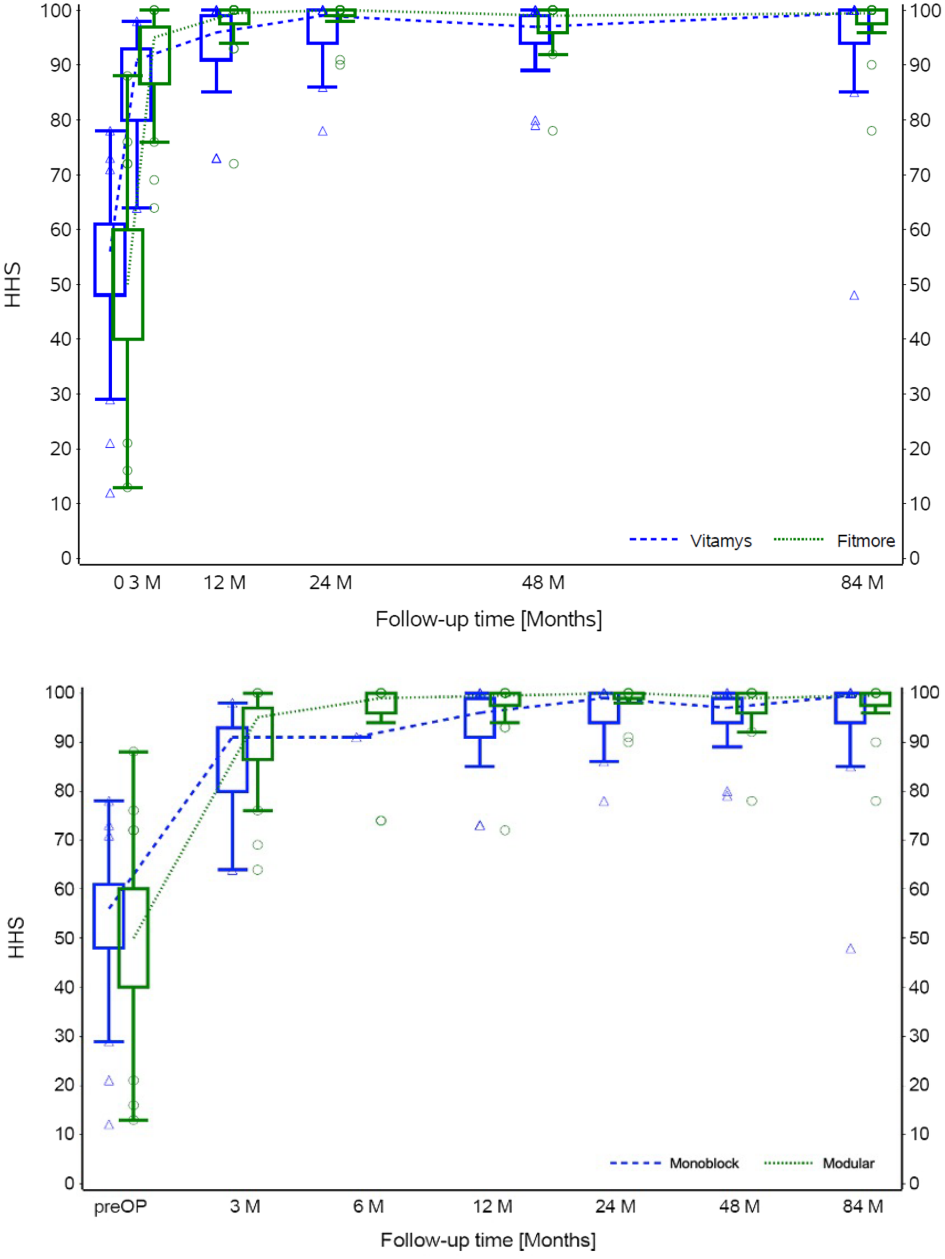
Box plot of the Harris Hip Score (HHS) over time

The HHS improved significantly in both groups without showing significant differences between the groups at the last follow-up (*p* = 0.425; Fig. 3).

No intraoperative complications were recorded. At the last follow-up, no revision surgery was needed, resulting in an implant survival rate of 100% in both groups. Heterotopic ossification (Brooker type 1) occurred once in each group.

In all patients, the components were implanted within the Lewinnek safe zone [17]. Mid-term assessments showed no evidence of osteolysis, lucent lines, or stress shielding. No further radiographic alterations were detected.

A total of 323 out of 391 radiographs were available for the EBRA analyses. The mean number of radiographs per patient was 4.0 ± 0.8 (range: 2–5). In the monoblock group, the mean cup migration at the mid-term follow-up was 1.67 ± 0.92 mm (range: 0.46–3.94 mm), while the annual migration rate decreased from 0.78 ± 0.58 mm/year (range: 0.24–1.99 mm/year) at 12 months to 0.27 ± 0.16 mm/year (range: 0.07–0.62 mm/year) at 5 years. In the modular group, the mean cup migration was 1.24 ± 0.87 mm (range: 0.22–3.62 mm). This group also showed a reduction in the annual migration rate from 0.89 ± 0.85 mm/year (range: 0.08–2.90 mm/year) at 12 months to 0.24 ± 0.15 mm/year (range: 0.03–0.54 mm/year) at 5 years. The annual migration rate appears to be comparable without statistically significant differences (*p* = 0.741).

In the first postoperative year, the mean wear in the monoblock group was 0.21 ± 0.18 mm (range: 0.00–0.70 mm). Mean total wear at the mid-term follow-up was 0.37 ± 0.28 mm (range: 0.05–1.42 mm), and the mean annual wear rate was 0.06 ± 0.04 mm/year (range: 0.01–0.22 mm/year). In the modular group, the mean wear was 0.20 ± 0.13 mm (range: 0.00–0.50 mm). The mean total wear at the mid-term follow-up was 0.35 ± 0.24 mm (range: 0.00–1.03 mm), and the mean annual wear rate was 0.07 ± 0.04 mm/year (range: 0.00–0.18 mm/year). No significant differences were observed in the annual wear rate between both cup types (*p* = 0.243).

## Discussion

The current study aimed to compare the clinical and radiological results and the EBRA measurements of wear and migration for a titanium-coated monoblock cup with VEPE and a modular metal-back cup with HXLPE. Both clinical and radiological results showed similar outcomes, and no significant differences were observed in wear rates and migration. No cup-related complications were observed at the mid-term follow-up, and none of the implants required revision surgery.

Encouraging survival rates have been previously reported for both cup designs investigated in the present study. The first-generation RM cups showed excellent long-term results for aseptic loosening with a survival rate of 94% after 20 years [18], while the vitamin-E-infused version, which has been available since 2009, also showed very good clinical and radiological mid-term results [19, 20]. While the vitamys cup has shown an excellent survival rate of 98.9% at 8-year and 9-month follow-up [20], long-term data for this cup design have not been published yet. The modular Fitmore cup has also demonstrated similarly excellent results, with a 10-year survival rate of 100% [21]. The findings for comparable modular cups, such as the Allofit press-fit cup (Zimmer Biomet, Warsaw, USA), are consistent with these results, with a survival rate of 98% after 11 years [22]. Thus, both cementless concepts have proven to be very successful.

Nevertheless, the data from the German Arthroplasty Register (EPRD) indicate that the modular cup remains the most frequently used cup type by far, accounting for 88% of all cases. In contrast, monoblock cups are used far less frequently, and accounted for only 9% of the cases in the annual report of 2022 [23]. Similarly, the modular cup remains the gold standard in Australia. In 2022, for the first time since 2003, a monoblock cup (RM Pressfit, Mathys) was one of the ten most used cementless cups in the Australian Orthopaedic Association National Joint Replacement Registry (AOANJRR) [24]. Additionally, the Swedish Arthroplasty Register (SAR) demonstrated a similar trend regarding acetabular component selection in its annual report of 2022 [25].

The lower usage rates of monoblock cups can be attributed to multiple reasons. The main purported disadvantage of this cup design is the lack of modularity, which rules out isolated liner exchange in cases involving early periprosthetic infections and after extended wear. Furthermore, assessment of proper cup seating due to the missing central screw hole is unfeasible with this cup design [26]. Coupled with the reasonable outcomes obtained using modular cups, these potential disadvantages of monoblock cups precluded their increased usage [21, 22]. On the other hand, however, issues related to long-term bone preservation after the use of modular cups have been reported and remain unresolved to date [6].

Brodt et al. [27] recently reported significantly less stress shielding using a monoblock implant in a randomized trial comparing the mid-term outcomes obtained with the RM Pressfit vitamys cup and the Allofit metal-backed modular cup (Zimmer Biomet, Warsaw, USA). Using dual-energy X-ray absorptiometry, they observed significantly less bone loss in the polar region of the RM Pressfit vitamys cup in comparison with the modular Allofit cup. In the monoblock polyethylene component, an elastic modulus closer to the human bone potentially provides better load transmission into the surrounding bone, whereas cementless rigid titanium cups seem to show considerable loss of periacetabular bone due to stress shielding during follow-up [6, 27].

Aseptic loosening of the acetabular component remains a major issue that can potentially necessitate revision surgeries [2, 4]. The 2022 annual report of the EPRD confirmed that the most common reason for reoperations was loosening (24.4%) [23]. Polyethylene wear is an indicator for aseptic loosening of implants caused by particle-induced osteolysis in the periacetabular bone [28]. Previous comparative studies have reported conflicting results in comparisons of wear rates between monoblock and modular cups. González Della Valle et al. found no difference in the wear rate between both cup types at 5.6 years [29], while Young et al. revealed a nonsignificantly lower wear rate and a significantly lower rate of osteolysis in monoblock cups in comparison with modular cups [30]. The authors explained that the higher wear rates of modular cups were attributable to backside wear [30], which has been recognized as an important contributor to liner wear in modular cups [5, 30].

Nevertheless, the proposed advantage of less backside wear in monoblock cups seems to be without clinical relevance, at least at mid-term follow-up. In the present study, the assessments of wear rate revealed similar results for both cup types. In this regard, the most substantial factor supposedly appears to be the wear of the articular surface, since backside wear has been found to show 1000-fold less effect on polyethylene wear than the wear of the articular surface [8, 31]. Dumbleton et al. [32] reported that osteolysis is rarely observed at wear rates of < 0.1 mm/year. Furthermore, a wear rate below 0.05 mm/year would eliminate the chance of osteolysis completely [32]. In the present study, the annual total wear rate was 0.06 mm in the monoblock group and 0.07 mm in the modular group. Therefore, both cups were clearly below the critical threshold without significant differences.

The latest refinement of polyethylene by adding vitamin E in HXLPE represents a milestone in improving the wear resistance and the longevity of the cup [14, 15, 33]. The antioxidant is added to prevent oxidation and improve the mechanical stability of polyethylene [13]. In vitro studies have reported increased oxidative stability of VEPE in comparison with HXLPE [14]. A recent meta-analysis and systematic review using radiostereometric analysis (RSA) by Zeng et al. also showed superior wear resistance of VEPE [33]. Rochcongar et al. obtained lower wear rates in the first 3 years with RM Pressfit vitamys cups in comparison with the previous RM Pressfit cup version with UHMWPE using RSA [34]. Several studies have reported the superiority of HXLPE to traditional UHMWPE with a reduced polyethylene wear rate and excellent long-term survival [33, 35, 36]. However, the mid-term results of the present study did not support the superiority of VEPE over HXLPE.

Early cup migration has been reported to be another major indicator for late aseptic loosening [37, 38]. The critical total cup migration threshold in EBRA measurements was defined by Krismer et al. as > 1 mm within the first 2 years [39]. Additionally, loosening was defined as an overall migration increase of 0.5 mm/year [40, 41]. However, the study by Stoeckl et al. [40] and other studies [42, 43] mentioned the critical threshold for total cup migration as > 2 mm within the first 2 years. Comparative studies analyzing the migration patterns between the monoblock and modular cups are rare. Baad-Hansen et al. [7], in a randomized RSA study, revealed no differences in terms of migration between both cup types. In the present study, the total cup migration of 0.94 mm was observed at 2 years in the monoblock group, which was below the abovementioned 1-mm threshold. In the modular group, the total cup migration at 2 years was 1.09 mm, which was slightly above the threshold reported by Krismer et al. [39]. However, at 5 years, the modular group showed a settlement with total cup migration of 1.24 mm without any signs of cup loosening. Thus, both cup types in the present study do not seem to be at high risk for late aseptic loosening. Furthermore, the results for both groups remained below the overall migration rate of 0.5 mm/year.

These results are backed up by the findings of a systematic review by Halma et al. [8], which also showed no difference in cup migration between monoblock and modular cups and no differences in implant survival when aseptic loosening was treated as the endpoint. However, the number of different implant properties and heterogeneous designs may be a confounding factor that limits the power of such reviews.

The present investigation had several limitations. The primary limitation was the relatively short follow-up time, particularly since wear and periacetabular bony alterations are known to be long-term issues. However, assessment of early alterations may allow for the prediction of late aseptic loosening. Another limitation was the retrospective study design. A randomized prospective study would be preferable. In addition, matched-pair studies have intrinsic limitations regarding validity, which also represents a limitation. Furthermore, the measurement method for wear and migration is a limitation. EBRA is a two-dimensional measurement software with limited accuracy but is well established for determining cup migration and wear rates as described by Krismer et al. [44]. The accuracy of EBRA measurements is 0.11 mm for wear and < 1 mm for migration [7, 8]. Therefore, assessments using EBRA should be performed with highly comparable radiographs, otherwise the measurements cannot be performed and will be excluded. Furthermore, the measurements were performed by a single observer to avoid inter-observer deviation. However, intra-observer deviation still remains. Another limitation was the use of different stems, which could potentially result in a relevant bias, especially in relation to the clinical results.

## Conclusion

The mid-term results obtained in this study support the equivalence of the investigated isoelastic press-fit monoblock VEPE cup with a modular metal-back HXLPE cup in terms of wear and cup migration at mid-term. Additionally, the two groups showed no obvious difference in clinical outcomes. Long-term studies are required to determine the effects of modularity, isoelasticity, and polyethylene stabilization with vitamin E on cup loosening and survival rates.

## Data Availability

The dataset generated and/or analysed during the current study are not publicly available due to the high volume of data but are available from the corresponding author on reasonable request.

## References

[1] Kurtz S, Lau E, Ong K (2009). Future young patient demand for primary and revision joint replacement: national projections from 2010 to 2030. Clin Orthop Relat Res.

[2] Kini SG, Anwar R, Bruce W, Walker P (2014). Modular versus monoblock cementless acetabular cups in primary total hip arthroplasty-a review. Int J Orthop (Hong Kong).

[3] Abu-Amer Y, Darwech I, Clohisy JC (2007). Aseptic loosening of total joint replacements: mechanisms underlying osteolysis and potential therapies. Arthr Res Ther.

[4] Sadoghi P, Liebensteiner M, Agreiter M (2013). Revision surgery after total joint arthroplasty: a complication-based analysis using worldwide arthroplasty registers. J Arthroplasty.

[5] Braun S, Sonntag R, Schroeder S (2019). Backside wear in acetabular hip joint replacement. Acta Biomater.

[6] Pitto RP, Bhargava A, Pandit S, Munro JT (2008). Retroacetabular stress-shielding in THA. Clin Orthop Relat Res.

[7] Baad-Hansen T, Kold S, Nielsen PT (2011). Comparison of trabecular metal cups and titanium fiber-mesh cups in primary hip arthroplasty: a randomized RSA and bone mineral densitometry study of 50 hips. Acta Orthop.

[8] Halma JJ, Vogely HC, Dhert WJ (2013). Do monoblock cups improve survivorship, decrease wear, or reduce osteolysis in uncemented total hip arthroplasty?. Clin Orthop Relat Res.

[9] Meneghini RM, Ford KS, McCollough CH (2010). Bone remodeling around porous metal cementless acetabular components. J Arthroplasty.

[10] Devane PA, Horne JG, Ashmore A (2017). Highly cross-linked polyethylene reduces wear and revision rates in total hip arthroplasty: A 10-year double-blinded randomized controlled trial. J Bone Joint Surg Am.

[11] Hopper RH, Ho H, Sritulanondha S (2018). Otto Aufranc award: crosslinking reduces tha wear, osteolysis, and revision rates at 15-year followup compared with noncrosslinked polyethylene. Clin Orthop Relat Res.

[12] Oral E, Wannomae KK, Hawkins N (2004). Alpha-tocopherol-doped irradiated UHMWPE for high fatigue resistance and low wear. Biomaterials.

[13] Beck M, Delfosse D, Lerf R, Knahr K (2012). Oxidation Prevention with Vitamin E in a HXLPE Isoelastic Monoblock Pressfit Cup: Preliminary Results. Total Hip Arthroplasty.

[14] Bracco P, Oral E (2011). Vitamin E-stabilized UHMWPE for total joint implants: a review. Clin Orthop Relat Res.

[15] Oral E, Muratoglu OK (2011). Vitamin E diffused, highly crosslinked UHMWPE: a review. Int Orthop.

[16] DeLee J, Charnley J (1976). Radiological demarcation of cemented sockets in total hip replacement. Clin Orthop Relat Res.

[17] Lewinnek GE, Lewis JL, Tarr R (1978). Dislocations after total hip-replacement arthroplasties. J Bone Joint Surg Am.

[18] Ihle M, Mai S, Pfluger D, Siebert W (2008). The results of the titanium-coated RM acetabular component at 20 years: a long-term follow-up of an uncemented primary total hip replacement. J Bone Joint Surg Br.

[19] Afghanyar Y, Joser S, Tecle J (2021). The concept of a cementless isoelastic monoblock cup made of highly cross-linked polyethylene infused with vitamin E: radiological analyses of migration and wear using EBRA and clinical outcomes at mid-term follow-up. BMC Musculoskelet Disord.

[20] Mahmood FF, Beck M, de Gast A (2021). Survivorship and patient-reported outcomes of an uncemented vitamin E-infused monoblock acetabular cup: a multicenter prospective cohort study. J Arthroplasty.

[21] Marchetti P, Binazzi R, Vaccari V (2005). Long-term results with cementless Fitek (or Fitmore) cups. J Arthroplasty.

[22] Streit MR, Weiss S, Andreas F (2014). 10-year results of the uncemented Allofit press-fit cup in young patients: 121 hips followed for 10–12 years. Acta Orthop.

[23] (2022) The German Arthroplasty Register (EPRD): Annual Report 2022. DGOOC. 10.36186/reporteprd072023

[24] (2022) Australian Orthopaedic Association National Joint Replacement Registry (AOANJRR). Hip, Knee & Shoulder Arthroplasty: 2022 Annual Report. AOA 1–487. https://aoanjrr.sahmri.com/annual-reports-2022]

[25] (2022) The Swedish Arthroplasty Register (SAR): Annual report 2022

[26] Weiss RJ, Hailer NP, Stark A, Kärrholm J (2012). Survival of uncemented acetabular monoblock cups: evaluation of 210 hips in the Swedish Hip Arthroplasty Register. Acta Orthop.

[27] Brodt S, Jacob B, Nowack D (2021). An isoelastic monoblock cup retains more acetabular and femoral bone than a modular press-fit cup: a prospective randomized controlled trial. J Bone Joint Surg Am.

[28] Comtesse S, de Gast A, Rehbein P (2020). Wear and migration are not influenced by head size in a vitamin E-infused highly cross-linked polyethylene acetabular cup. Orthop Traumatol Surg Res.

[29] González Della Valle A, Su E, Zoppi A (2004). Wear and periprosthetic osteolysis in a match-paired study of modular and nonmodular uncemented acetabular cups. J Arthroplasty.

[30] Young AM, Sychterz CJ, Hopper RH, Engh CA (2002). Effect of acetabular modularity on polyethylene wear and osteolysis in total hip arthroplasty. J Bone Joint Surg Am.

[31] Kurtz SM, Ochoa JA, Hovey CB, White CV (1999). Simulation of initial frontside and backside wear rates in a modular acetabular component with multiple screw holes. J Biomech.

[32] Dumbleton JH, Manley MT, Edidin AA (2002). A literature review of the association between wear rate and osteolysis in total hip arthroplasty. J Arthroplasty.

[33] Li Z, Xiang S, Wu C (2021). Vitamin E highly cross-linked polyethylene reduces mid-term wear in primary total hip replacement: a meta-analysis and systematic review of randomized clinical trials using radiostereometric analysis. EFORT Open Rev.

[34] Rochcongar G, Buia G, Bourroux E (2018). Creep and wear in Vitamin E-infused highly cross-linked polyethylene cups for total hip arthroplasty: a prospective randomized controlled trial. J Bone Joint Surg Am.

[35] Engh CA, Hopper RH, Huynh C (2012). A prospective, randomized study of cross-linked and non–cross-linked polyethylene for total hip arthroplasty at 10-year follow-up. J Arthroplasty.

[36] Langlois J, Atlan F, Scemama C (2015). A randomised controlled trial comparing highly cross-linked and contemporary annealed polyethylene after a minimal eight-year follow-up in total hip arthroplasty using cemented acetabular components. Bone Joint J.

[37] Stocks G, Freeman M, Evans S (1995). Acetabular cup migration. Prediction of aseptic loosening. J Bone Joint Surg British.

[38] Streit MR, Haeussler D, Bruckner T (2016). Early migration predicts aseptic loosening of cementless femoral stems: a long-term study. Clin Orthop Relat Res.

[39] Krismer M, Stöckl B, Fischer M (1996). Early migration predicts late aseptic failure of hip sockets. J Bone Joint Surg British.

[40] Stoeckl B, Brabec E, Wanner S (2005). Radiographic evaluation of the Duraloc cup after 4 years. Int Orthop.

[41] Wilkinson J, Hamer A, Elson R (2002). Precision of EBRA-Digital software for monitoring implant migration after total hip arthroplasty. J Arthroplasty.

[42] Wyatt M, Weidner J, Pfluger D, Beck M (2017). The RM Pressfit vitamys: 5-year Swiss experience of the first 100 cups. Hip Int.

[43] Afghanyar Y, Danckwardt C, Schwieger M (2020). Primary stability of calcar-guided short-stem total hip arthroplasty in the treatment of osteonecrosis of the femoral head: migration analysis using EBRA-FCA. Arch Orthop Trauma Surg.

[44] Krismer M, Bauer R, Tschupik J, Mayrhofer P (1995). EBRA: a method to measure migration of acetabular components. J Biomech.

